# Estimating the optimal efflux inhibitor concentration of carvacrol as a function of the bacterial physiological state

**DOI:** 10.3389/fmicb.2023.1073798

**Published:** 2023-01-25

**Authors:** Anna Jánosity, József Baranyi, Botond Bendegúz Surányi, Sonja Smole Možina, Andrea Taczman-Brückner, Gabriella Kiskó, Anja Klančnik

**Affiliations:** ^1^Department of Food Microbiology, Hygiene and Safety, Institute of Food Science and Technology, Hungarian University of Agriculture and Life Sciences, Budapest, Hungary; ^2^Department of Food Science and Technology, Biotechnical Faculty, University of Ljubljana, Ljubljana, Slovenia

**Keywords:** efflux inhibitor, carvacrol, physiological-state-dependent resistance, fluorescence-based assays, predictive modeling

## Abstract

Our aim was to find the optimal efflux inhibitor concentration of a natural component, carvacrol, as a function of the physiological state of *Escherichia coli*. Using fluorescence-based measurements with two strains of *E. coli*, the effect of carvacrol was assessed at 17 sub-inhibitory concentrations, at which the bacterial efflux mechanism was compromised. The efficacy of carvacrol, as an efflux inhibitor, was compared to synthetic inhibitors and we found carvacrol the most efficient one. We considered the accumulation of Ethidium Bromide (EtBr) as a proxy for drugs spreading in the cell, thus measuring the efflux activity indirectly. The change in membrane integrity caused by the exposure to carvacrol was monitored using the LIVE/DEAD BacLight Bacterial Viability kit. To find the optimal inhibitory concentration of carvacrol, we used predictive microbiology methods. This optimum varied with the bacterial physiological state, as non-growing cultures were less susceptible to the effect of carvacrol than growing cultures were. Moreover, we point out, for the first time, that the efflux-mediated resistance of untreated cultures was also stronger in the non-growing than in the growing phase at population level.

## 1. Introduction

Antibiotic resistance is a serious threat, against which restoring and enhancing the bacterial susceptibility to antibiotics, *via* the use of efflux inhibitors, could be a possible solution. Active efflux of drugs by transmembrane proteins has a positive effect on bacterial survival and may even play a role in the development of permanent resistance to those drugs (Walsh and Wencewicz, 2016). The search for small, efflux-inhibiting molecules is an active and rapidly growing research area. Such inhibitors, in a combined use, reduce the required dosage of chemically diverse agents, such as disinfectants, detergents, or antibiotics (Van Bambeke et al., 2006).

Bacterial efflux systems are membrane-embedded proteins, located in the outer membrane, within periplasm and in the cytoplasmic membrane of Gram-negative bacteria. These transmembrane proteins are responsible for the multiple drug resistance of microorganisms (Teelucksingh et al., 2020). Efflux pumps (EPs) can be found in all bacterial species and the coding genes are general, present in both antibiotic-susceptible and antibiotic-resistant bacteria (Piddock, 2006). A successful efflux inhibitor needs to be safe, non-toxic, and effective against a wide range of microorganisms (Bhardwaj and Mohanty, 2012). Even more importantly, the molecule itself, at the applied concentration, must not act as an antimicrobial (e.g., *via* damaging the membrane integrity), otherwise, in the long term, bacterial resistance could be induced against it. Besides, natural inhibitors, such as carvacrol, are not toxic, which is of high importance, as toxicity is the major problem with chemical inhibitors (Kourtesi et al., 2013; Ferrer-Espada et al., 2019).

The antimicrobial efficiency of natural compounds depends on the location of functional groups on their molecules, e.g., on the position of hydroxyl group on the phenolic rings (Nazzaro et al., 2013). Carvacrol, a monoterpene found in *Origanum vulgare*, *Thymus vulgaris*, and other plants, is already known for its antibacterial activity, as it affects the efflux defense system of cells (Boren et al., 2020; Wijesundara et al., 2021). It has been shown that carvacrol disrupts the cell membrane, *via* inhibiting the activity of ATPases. Aside from inhibiting those activities, carvacrol releases intracellular adenosine triphosphate and other cell components. It was found that in phenolic compounds (of which carvacrol is one), the presence of hydroxyl group and delocalized electrons would allow the compound to act as a proton exchanger (Ultee et al., 1999), thus reducing the gradient across the cytoplasmic membrane (Burt, 2004; Ben Arfa et al., 2006; Pisoschi et al., 2018). However, at high concentrations, carvacrol makes pores on the lipid bilayer of the cytoplasmic membrane (Guarda et al., 2011; Friedman, 2015), therefore its membrane degradation effect must be taken into consideration when applying it as an efflux inhibitor.

It is desirable to quantify the efficacy of efflux inhibitors for which multiple methods can be used (Blair and Piddock, 2016; Spengler et al., 2017) and those provide information about the EP activity of bacteria simultaneously. Probably the most well-known laboratory efflux pump inhibitors (EPIs) are carbonyl cyanide-m-chlorophenylhydrazone (CCCP), Phenylalanine-arginine β-naphthylamide (PaβN), and 1-(1Naphthylmethyl) piperazine (NMP). All of them are broad spectrum inhibitors, showing a potential activity against Gram-negative bacteria and could reduce the activity of AcrAB efflux system in Enterobacteriaceae family (Kumar et al., 2013). PaβN is considered as a competitive natured inhibitor. This means that while the pump extrudes PaβN out of the cells, the drug remains in the cells. In this way, the drug compound can reach the concentration required for its activity on the target (Pagès and Amaral, 2009). NMP was tested in several bacterial species such as in *Acinetobacter baumannii*, in different species of the Enterobacteriaceae family and in *E. coli*. NMP is considered to be one of the most potent compounds against *E. coli*. However, NMP, like all the listed compounds, has serious chronic health effects, thus potentially hazardous to humans (Mahamoud et al., 2007; Marchetti et al., 2012).

Intrinsic (i.e., not acquired but genetically coded) resistance of bacteria to drugs has been found to be growth-phase-dependent (Kobayashi et al., 2006; Greulich et al., 2015; Smirnova and Oktyabrsky, 2018) lending itself to the question: how does the efflux mechanism and its inhibition depend on the physiological state of the bacterial culture?

To answer this question, here we turn to predictive microbiology methods. The main objective of this discipline is to quantitatively describe the effect of external factors, e.g., temperature, pH or water activity, on the bacterial behavior in food-related environments. Recently, such quantitative methods were used to characterize the response of *E. coli* to carvacrol therapy (Jánosity et al., 2021). In that study, following the terminology established in predictive microbiology, *primary models* described the temporal changes of the fluorescence signal (*Fs*), which was an indicator how EtBr spread in the cells. *Secondary models* were used to analyze the effect of carvacrol, at sub-inhibitory concentrations, on the rate of that spread.

In the present work, we add new results to this analysis and show that the optimal concentration of carvacrol depends on the physiological state of the organism. Moreover, we compare its EPI efficacy with that of two synthetic inhibitors.

## 2. Materials and methods

### 2.1. Bacterial strains, growth conditions, OD growth curves

Two strains of *E. coli* were used to study their bacterial efflux: ŽM 370 (ATCC 11229) is a pathogenic reference strain while *E. coli* ŽM 513 (VF 3584) was isolated from steak tartare; both isolates were from the Veterinary Faculty of the University of Ljubljana. Stock cultures of these strains were stored at −80°C, then sub-cultured twice and maintained on Tryptic Soy Agar (Oxoid, Basingstoke, Hampshire, United Kingdom). Overnight cultures were prepared in the third passage inoculated in Tryptic Soy Broth (Oxoid, Basingstoke, Hampshire, UK), and incubated for 24 h, without carvacrol at 37°C.

The overnight cultures had been diluted in fresh Tryptic Soy Broth at OD_600_ = 0.1 before the fluorescent assays. Three physiological states were tested: fast-, slow-, and non-growing phases. To achieve them, freshly diluted cell cultures were incubated at 37°C for 0.5 h, 4 h, and 12–16 h, respectively. The final test cultures used in fluorescence assays had the same concentrations (OD_600_ = 0.2). The incubation times were decided according to the growth curves measured at 600 nm and 37°C using a Tecan Safire 2 microplate reader (Tecan, Zürich, Switzerland).

### 2.2. Natural compound and chemicals

We used Ethidium Bromide (EtBr) to measure the efflux activity indirectly and the efflux modulating effect of carvacrol (Blair and Piddock, 2016). EtBr, a fluorescence dye, simulated drug accumulation inside the bacterial cells.

Carvacrol, our chosen natural compound with a purity of ≥98%, and EtBr, the efflux substrate, were derived from Sigma-Aldrich Chemie, Steinheim, Germany. Carvacrol stock solutions were prepared in absolute ethanol of 100 mM (15.022 mg/ml) concentration. NMP and PAβN were tested as synthetic efflux inhibitors and obtained from CHESS GmbH (Mannheim, Germany). NMP stock solution was prepared in EtOH and stock solution of PaβN in sterile water as in 20 mg/l concentration. LIVE/DEAD BacLight Bacterial Viability Kit (L-7012; Molecular Probes, Eugene, Oregon, United States) measured the changes of membrane permeability. To wash the cultures and resuspend for fluorescent probes, Phosphate Buffered Saline (PBS) tablets were used from Oxoid (Basingstoke, Hampshire, United Kingdom).

### 2.3. OD growth curves to determine physiological states

The OD growth curves of microorganisms were measured in Tryptic Soy Broth at 37°C, with the initial inoculum level of OD_600_ = 0.1 (≈10^8^ CFU/ml). Turbidity of wells was measured at 600 nm, using a Tecan Safire 2 microplate reader (Tecan, Zürich, Switzerland). Experiments were performed in 96-well microtiter plates, with the final volume of 100 μl *per* well, in triplicates.

### 2.4. Culture preparation before fluorescence-based assays

The kinetics of EtBr accumulation and the rate of membrane degradation were measured (Kovač et al., 2015) by a VarioskanLUX multimode microplate reader (Thermo Fisher Scientific, Waltham, Massachusetts, United States). Culture preparations and treatments were the same before both types of assays. Following the incubation, cell cultures from physiological states were washed and resuspended in PBS, then diluted to OD_600_ = 0.2 which is equivalent to *ca.* 10^9^ CFU/ml cell concentration. The procedure allowed to set the cell concentrations to be the same for the cultures in different physiological states. Carvacrol was then added to the dilution. We quantified this treatment by the ratio between the carvacrol concentration and its MIC (minimum inhibitory concentration) value. The 17 test-concentrations were chosen from a sub-inhibitory range, between 0.1 and 0.5 MIC, which corresponds to 30 mg/l and 150 mg/l of carvacrol. The interval was divided equidistantly by step of 0.025 MIC. Similarly, the efflux modulation effect of NMP was tested in 0, 100, 200 and 300 mg/l concentrations and efflux inhibition activity of PaβN was tested only in 22 mg/l concentration as it was suggested by Kurinčič et al. (2012). Finally, fluorescent dyes (EtBr or LIVE/DEAD BacLight Bacterial Viability kit) were added to both the treated and the non-treated cultures. These tests were carried out in three dependent (parallel) and two independent replicates using black plates.

### 2.5. Settings of EtBr accumulation assays

The EtBr accumulation inside the bacterial cell was measured according to our previous study (Jánosity et al., 2021), by relative fluorescence unit (RFU). Readings were made in 45 s intervals over 1 h observation time.

### 2.6. Settings of membrane integrity assays

The membrane integrity of bacteria was measured by LIVE/DEAD BacLight Bacterial Viability kit, as a mixture of green-fluorescent dye SYTO 9 and propidium iodide (Berney et al., 2007). The basis of this technique is that while green dye can pass through damaged and undamaged cell membranes, red dye can only pass through damaged membranes. The stability of cell membrane was determined by the reduction of the green fluorescence signal. In the presence of red dye, the fluorescence signal from green dye decreases. The intracellular propidium iodide penetration measures the cytoplasmic membrane damage, which was quantified by RFU values, with reference to SYTO 9 fluorescence at λ_ex_ = 481 nm and λ_em_ = 510 nm. Readings were made in 60 s intervals over 1 h. As a negative control, the membrane integrity of heat-treated culture (at 80°C for 15 min) was also measured.

### 2.7. Numerical and statistical methods

EtBr accumulation and membrane integrity changes of microorganisms were analyzed according to the modeling approach of Jánosity et al. (2021) that we summarize here.

#### 2.7.1. Primary models

At each carvacrol concentration, the temporal variation of *Fs* values, in the unit of RFU, was measured and fitted by primary models: (A) saturation model of EtBr accumulation (Equation 1) or (B) a dissipation model of membrane integrity (Equation 2):


(1) $$Fs\left(t\right)=Fs_{0}+\left(Fs_{\max}-Fs_{0}\right)\cdot \left(1-e^{r\cdot t}\right)+\varepsilon$$

(2) $$Fs\left(t\right)=Fs_{0}-\left(Fs_{0}-Fs_{\min}\right)\cdot \left(1-e^{r\cdot t}\right)+\varepsilon$$

Here, Fs(t) is the Fs value at the time *t* elapsed from an initial time $t_{0}$; $Fs_{0}$ is its value at the initial time; $Fs_{\max}$ is its theoretical (asymptotic) maximum; $Fs_{\min}$ is its theoretical minimum; and *r* is the exponential rate at which the Fs(t) function converges to $Fs_{\max}$ or $Fs_{\min}$, depending on the type of fluorescent assay, described by the primary models, finally, ε is a random measurement error.

#### 2.7.2. Secondary models

The ratio between the highest ($Fs_{\max}$) and lowest ($Fs_{0}$) fitted *Fs* values was chosen to quantify the efficacy of carvacrol as efflux inhibitor (secondary model). As the fitted $Fs_{\max}$ values were frequently far from the measured data, this parameter was replaced, for practical purposes, by the maximum of those data, denoted by $Fs_{1}$= Fs(1) in what follows. The $Fs_{1}$/$Fs_{0}$ ratio is the factor by which the Fs values increased during the [0,1] observation time. The variation of the natural logarithm of this parameter, as a function of the carvacrol concentration, x, was modelled for each strain and physiological state, by an asymmetric, convex-from-below, bi-linear (triangle-) function denoted by $B_{s}$ (Equation 3):


(3) $$y_{EP}\left(x\right)=\ln\frac{Fs_{1}}{Fs_{0}}=B_{s}\left(x\right)=y_{opt}\cdot \left\{\begin{array}{c} \;\frac{\left(x-x_{\min}\right)}{\left(x_{opt}-x_{\min}\right)}\;\left(x_{\min}\leq x\leq x_{opt}\right)\\ \frac{\left(x_{\max}-x\right)}{\left(x_{\max}-x_{opt}\right)}\;\left(x_{opt}\leq x\leq x_{\max}\right) \end{array}\right.\;$$

The $x_{\min}$, $x_{opt}$, $x_{\max}$ parameters are the minimum, optimum, and maximum concentrations defining the bi-linear function. Note that, in this range the $\frac{Fs_{1}}{Fs_{0}}$ factor was greater than 1 (i.e., the Fs values increased during the experiment). The scaling constant $y_{opt}$ is the value of this factor at the optimum carvacrol concentration. The s in the index of the $B_{s}$ notation indicates that we expect the bi-linear function to depend on the physiological state of the culture. Outside the [$x_{\min}$, $x_{\max}$] interval, we assume that the Fs values do not grow during the observation time, i.e., the $B_{s}$- function is zero there.

Non-linear regression and F-test were used to determine the optimum inhibitory concentrations of carvacrol and the significance of this parameter at α = 0.05 level.

## 3. Results

### 3.1. OD curves and determining the physiological states

**Figure 1:**
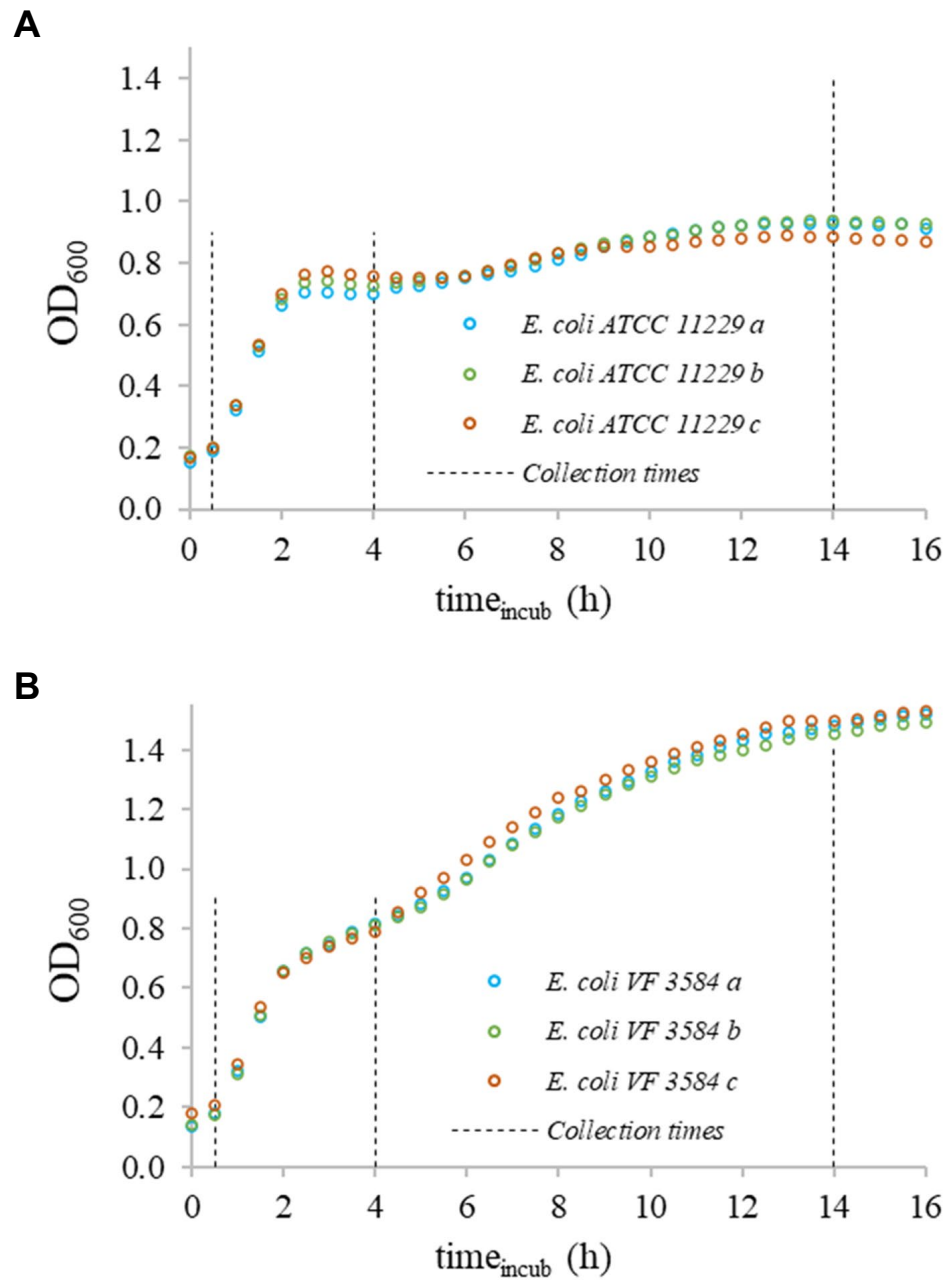
OD_600_ growth curves of bacteria in triplicates recorded at 37°C. Uppercase letters indicate the different strains: **(A)** *Escherichia coli* ATCC 11229 and **(B)** *E. coli* VF 3584; lowercase letters specify the three replicates. The starting concentration of suspensions was ≈10^8^  CFU/ml. Data points were shifted to an initial OD_600_ = 0.1 value for all curves.

Prior to fluorescent assays, *E. coli* cells were collected at 0.5, 4, and 12–16 h after incubation at 37°C (Figure 1).

**Figure 2:**
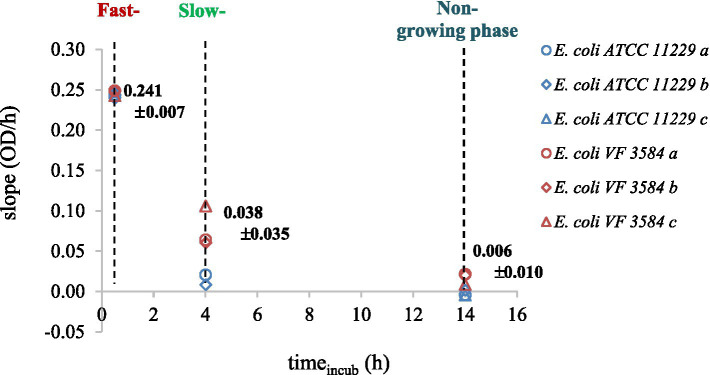
Physiological states of *E. coli* ATCC 11229 (blue markers) and *E. coli* VF 3584 (red markers) were quantified by the slopes of the OD-curves. Measurements were carried out in triplicates noted by *a*, *b*, and *c* letters.

The slopes of the recorded OD-curves were used to determine the fast-, slow-, and non-growing phases of cultures. The different physiological states were identified after 0.5, 4, and 14 h incubation (Figure 2).

### 3.2. The minimum inhibitory concentration of carvacrol and its influence on the fluorescence signal

**Figure 3:**
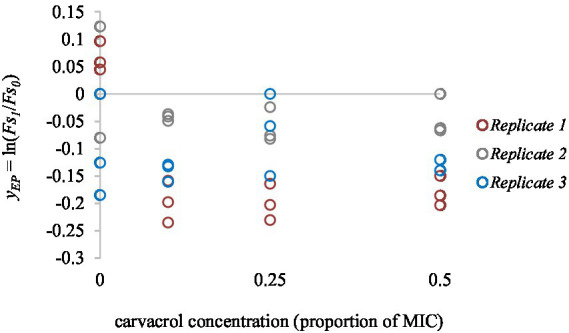
The concentration dependent effect of carvacrol on EtBr fluorescent signal intensity. Carvacrol was tested between 0 and 0.5 MIC values (0, 25, 75, and 150 mg/l concentrations). Colors indicate the independent replicates.

Carvacrol showed compelling antimicrobial effects. Our previous study established its MIC value at 300 mg/l for both strains. As a negative control, we measured the effect of carvacrol on $F_{S}$ when it was added to blank, only PBS containing wells. We found no significant difference between the 0.1, 0.25, and 0.5 MIC treatments (*p* = 0.56). However, carvacrol has lowered the fluorescence signal itself, which was taken into account when the non-treated cultures were analyzed (Figure 3).

### 3.3. Primary model of efflux inhibition and membrane integrity

**Figure 4:**
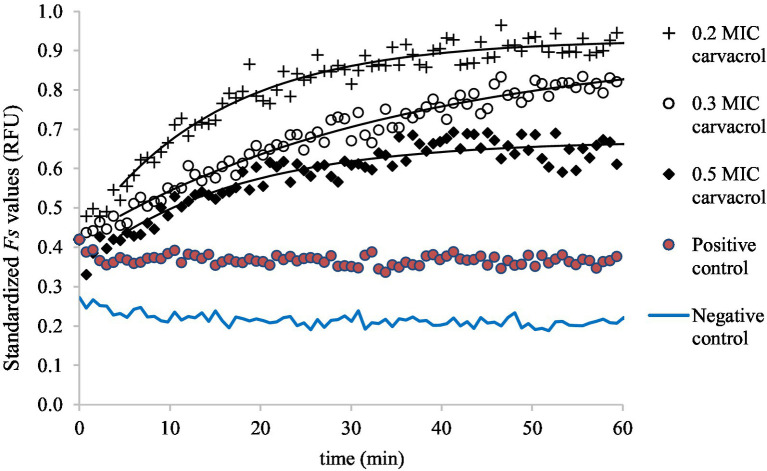
Saturation model fitted to the standardized *Fs* curves, demonstrating the accumulation of EtBr at 0.2, 0.3, and 0.5 MIC carvacrol treatments, in slow-growing culture of *E. coli* ATCC 11229. As a negative control, the *Fs* values were measured in uninoculated suspension (in PBS) while the non-treated culture was the positive control.

**Figure 5:**
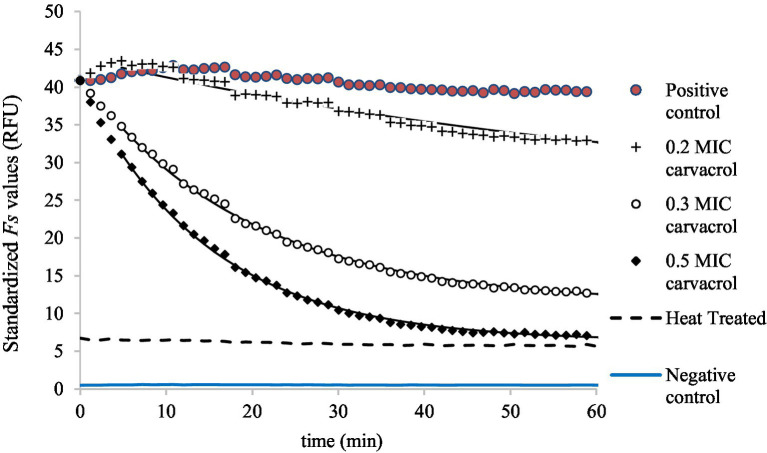
Dissipation model fitted to the standardized *Fs* curves, demonstrating the membrane integrity of bacteria at 0.2, 0.3, and 0.5 MIC carvacrol treatments in exponential phase culture of *E. coli* ATCC 11229. As a negative control, *Fs* values were also measured in uninoculated suspension (PBS) and membrane integrity of heat-treated culture (80°C, 15 min) is also shown. Non-treated culture was the positive control.

The zero time for the primary models were set to *t_0_* = 0.08 h (≈ 5 min) for all curves to avoid the initial noise. For better visualization, the carvacrol-treated *Fs* curves are shifted to a common starting point with non-treated *Fs* curves for clarity and easier comparison (Figures 4, 5).

Figure 3 shows the EtBr accumulation in *E. coli* ATCC 11229, for slow-growing inocula. The applied carvacrol treatments were 0.2, 0.3, and 0.5 MIC values (which correspond to 60, 90, and 150 mg/l concentrations). The results of the positive (non-treated culture) and negative (uninoculated sample) controls are also shown to demonstrate that even low concentrations of carvacrol improved the EtBr accumulation rate. The biggest total EtBr accumulation was detected at 0.2 MIC of carvacrol treatment.

Symbols represent the experimental values and the black continuous lines show the saturation models fitted to the temporal curves. In the absence of carvacrol treatment, the Fs(t) fluorescent signals can be described as constant values though the $Fs_{0}$ and the $Fs_{max}$ parameters increased with the amount of added carvacrol.

Membrane integrity was measured similarly to EtBr accumulation (Figure 5). As carvacrol increased, so decreased the membrane integrity of bacteria; this is why the primary model was chosen to be a mirror image of the saturation curve: the Fs(t) function converges to an $Fs_{min}$ value.

### 3.4. Secondary model of efflux inhibition

In our previous study (Jánosity et al., 2021), we found that a bi-linear fit represents a significant improvement compared to a linear one to describe the efflux inhibition as a function of carvacrol treatment. The convex-from-below model indicated the existence of an optimum. The optimum refers to the concentration of carvacrol where the efflux inhibition is the highest. At these points the $\ln\;(Fs_{1}/Fs_{0})$ ratio reached its highest values, indicating maximum EtBr accumulation rate.

**Figure 6:**
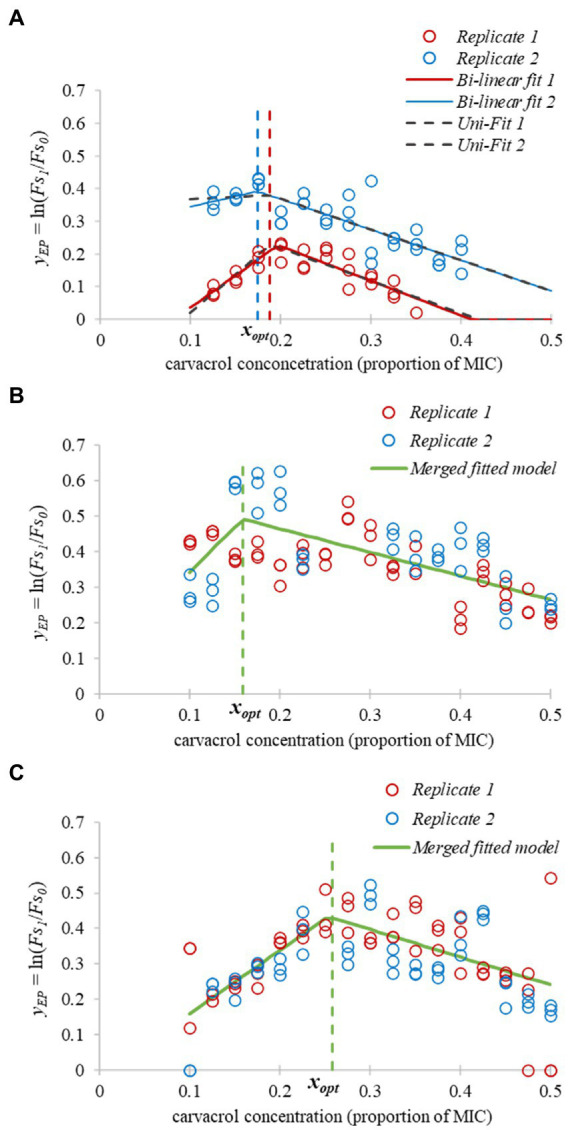
Bi-linear functions as secondary models for efflux inhibition. The chosen parameter of the primary model fitting was $y_{EP}=\ln\;(Fs_{1}/Fs_{0})$, measuring the influence of carvacrol on the efflux mechanism *via* EtBr accumulation. Numbers indicate physiological states; **(A)** – fast-growing, **(B)** – slow-growing, and **(C)** – non-growing cultures of *E. coli* ATCC 11229. The $x_{opt}$ refers to the optimum concentration of carvacrol, where the efflux inhibition is the highest.

For both strains we generated six data sets (three physiological states, two independent replicates). Results of *E. coli* ATCC 11229 are shown on Figure 6.

If the primary model showed $y_{EP}$<0.01, indicating that the increase of the Fs values was less than 1%, then these measurements were omitted. In some cases, the Fs values were obvious outliers at the edges of the [0.1, 0.5 MIC] region of interest for the carvacrol concentration, for example due to excessive damage of the cell membrane. In such cases we narrowed the region of interest, but never down to an interval smaller than [0.125, 0.45 MIC]. Out of the 12 regressions, we could not justify the use of the bi-linear function in two cases, because it did not provide significant improvement from the linear fit and these experiments were omitted from further analysis.

### 3.5. Secondary model of membrane integrity changes

**Figure 7:**
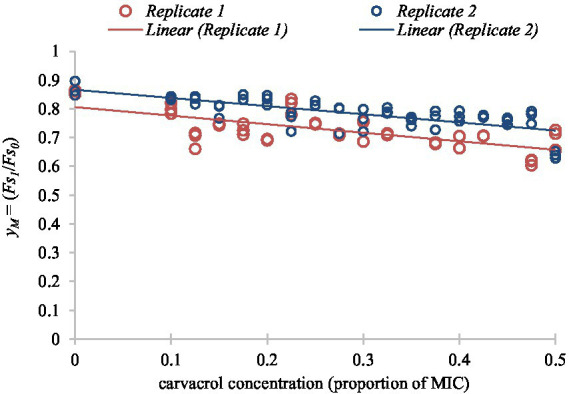
Linear function fitted to the secondary model of membrane integrity readings according to different concentrations of carvacrol for non-growing cultures of *E. coli* VF 3584.

The primary model parameter of membrane integrity measurements was determined similarly to the previous procedure; but simply the $y_{M}=(Fs_{1}/Fs_{0})$ ratio was used to quantify the membrane damage caused by carvacrol. The secondary model of membrane integrity measurements was a monotone function of carvacrol, without an optimum: the higher the carvacrol concentration, the lower the membrane integrity (Figure 7).

To express the membrane degradation effect of carvacrol in percentage, $y_{M}$ values were multiplied by 100 and corrected with the average relative decrease/increase of Fs. It was calculated from the fitted points with the assumption that the membrane integrity of the non-treated cells should be 100%.

### 3.6. Dependence of secondary model parameters on the physiological state of the culture

We studied how the secondary model parameters of EtBr accumulation (maximum point of $y_{EP}$ values) depend on the physiological state of bacteria. The optimum efflux inhibitory concentrations of carvacrol ($x_{opt}$) values are shown in Table 1. We found that the optimum depended (*p* < 0.05) on whether the inoculum was from growing or non-growing culture. The strain-variability was not significant, except for non-growing cultures.

**Table 1:** Optimum efflux inhibitor concentrations of carvacrol against two *Escherichia coli* strains and the associated membrane damages (%) as a function of the bacterial physiological states.

	*E. coli* ATCC 11229		*E. coli* VF 3584	
	Optimum efflux inhibitor concentration of carvacrol (proportion of MIC)	Membrane damage	Optimum efflux inhibitor concentration of carvacrol (proportion of MIC)	Membrane damage
Fast-growing phase	0.185 ± 0.008 *	22%	0.149 ± 0.014	10%
Slow-growing phase	0.159 ± 0.010	21%	0.175 ± 0.009	18%
**Non-growing phase**	0.254 ± 0.009	18%	0.311 ± 0.007*	11%

Carvacrol concentrations are expressed as proportions of MIC = 300 mg/l.

*Asterix shows where the optimum inhibitor concentrations for the two strains are significantly different (*p* < 0.05).

Table 1 shows the results of the regression of the secondary model, which was carried out on two independent replicate datasets. F-test was used to decide whether the two datasets can be merged. The optimum carvacrol concentration was estimated by the location of the breakpoint of the bi-linear function (secondary model) fitted to the primary model parameter in question. In certain cases, denoted by *, the two replicate datasets could not be merged, based on an F-test; however, even in those cases, the optimum carvacrol values for the two replicates did not differ significantly (*p* > 0.05).

Table 1, as well as Figure 6, demonstrate that the highest optimum carvacrol concentrations to inhibit the efflux of bacteria are obtained for the non-growing cultures. This result confirms our expectation that the bacterial resistance mechanism is weaker in the fast- and slow-growing phases than in the non-growing phase. Namely, in the first two cases, lower carvacrol concentrations were enough to reach the greatest efflux inhibition.

### 3.7. Effect of physiological state on the bacterial efflux activity in the absence of carvacrol

**Figure 8:**
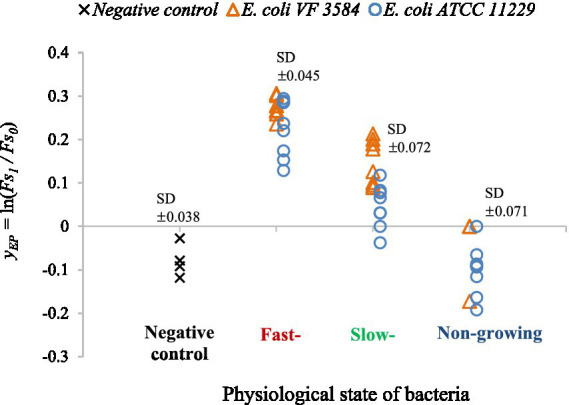
*y*_*EP*_ values of non-treated cultures to describe the efflux activity as a function of the physiological state of the cells. Untreated cultures of *E. coli* VF 3584 (orange markers) and *E. coli* ATCC 11229 (blue markers) were tested for fast-, slow- and non-growing phases. As a negative control the *y*_*EP*_ measured in PBS are also shown (black markers).

To describe the growth history-related efflux mechanism of the non-treated *E. coli* cultures the total variation of $y_{EP}$ values (primary model parameters of EtBr accumulation) was studied (Figure 8). The physiological state was divided into the three categories: fast-, slow-, and non-growing phases. Data of uninoculated suspension (EtBr accumulation measured in PBS - negative control) were also generated.

The observations suggested that the efflux activity of bacteria strongly depended on the growth history of cultures. In non-growing phase, the $y_{EP}$ values showed a pattern similar to the negative control, only the noise is bigger in the former case. This means that EtBr could not accumulate in these bacteria as we found no increase between the $F_{S0}$ and $F_{Smax}$ values in the one-hour-long observation interval. This suggests a strong intrinsic resistance for the non-growing culture. In growing phases bacteria are focusing on adaptation and replication while the efflux mechanism seemed to be weaker. In these phases, the $y_{EP}$ levels were greater than in the non-growing phase, so cells could not extrude completely the EtBr which can explain our figure.

In the non-growing phase, the resistance mechanism of bacteria is well developed, bacterial cell wall can become less permeable compared to the other two phases and the cells are more stressed (Jananee and Preeti, 2017). The results are consistent with data in Table 1, where we showed that the optimal efflux inhibitor concentrations of carvacrol are significantly lower in fast- and slow-growing phases than in the non-growing phase. To summarize all the above, we found that the efflux of non-treated bacteria is weaker in the first two physiological states, furthermore lower carvacrol concentrations were sufficient to achieve the highest efflux inhibition in those physiological states.

### 3.8. Efflux modulation activity of synthetic inhibitors

**Figure 9:**
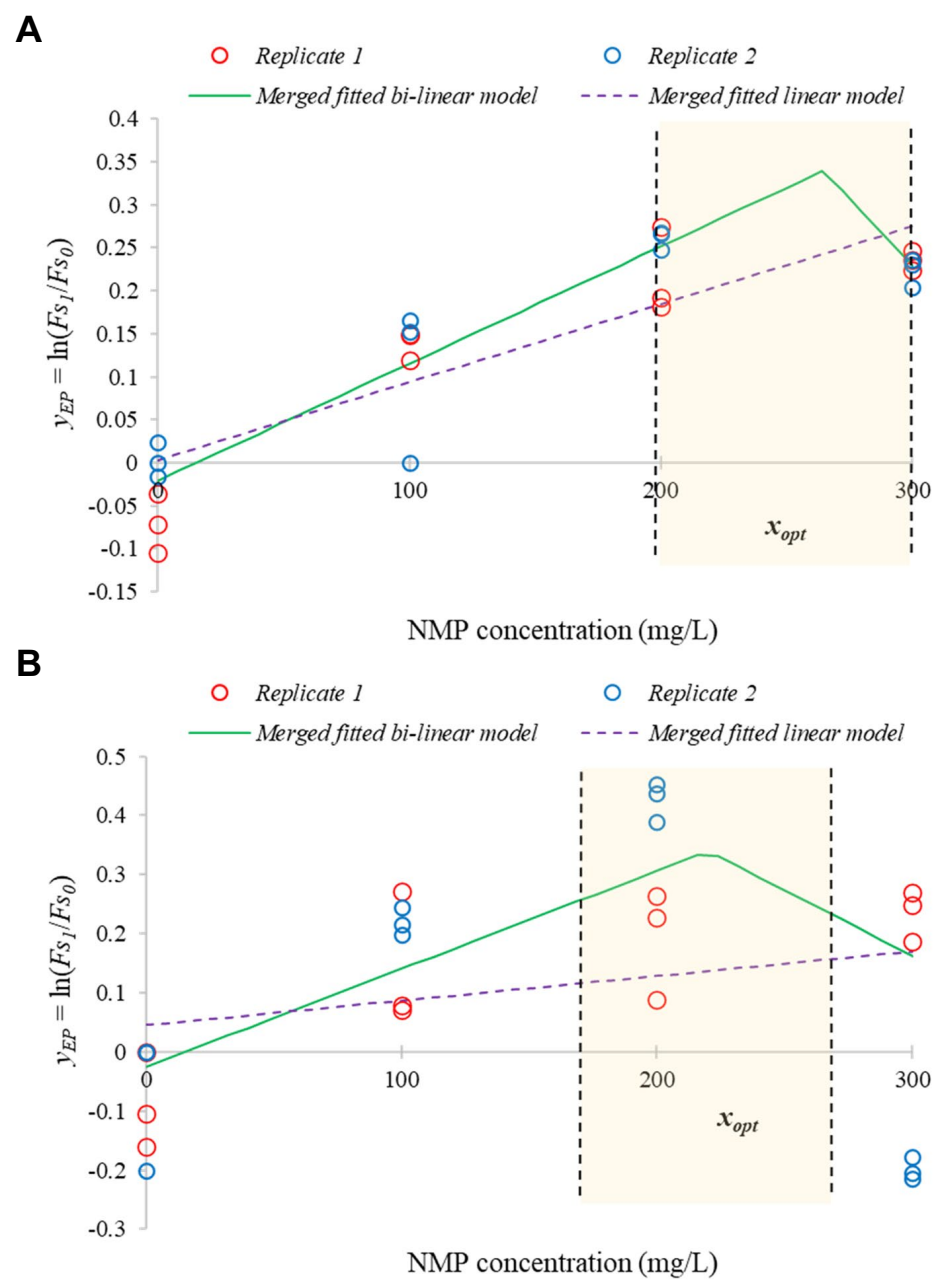
Bi-linear secondary models to describe the efflux inhibition by NMP, a synthetic inhibitor. The fit determines the range of optimum NMP concentration. The bi-linear function (continuous green lines) significantly improves the fit compared to the linear function (dashed purple lines). The figure is based on two independent replicates using stationary phase cultures of *E. coli* VF 3584 **(A)** and *E. coli* ATCC 11229 **(B)**.

We also measured and evaluated the efflux modulation activity as a function of NMP concentration. EtBr accumulation was measured when 0, 100, 200, and 300 mg/l NMP was added to the cultures, while the bacteria were in stationary phase. As a primary model, the saturation function was suitable to describe the temporal variation of the Fs values obtained by EtBr accumulation assays. The bi-linear secondary model, describing the $y_{EP}$ parameter as a function of the NMP treatment, described the EP inhibition significantly better than the linear model (*F* = 5.295, *p* = 0.012 for *E. coli* ATCC 11229 and *F* = 14.908, *p* = 7*10^−5^ for *E. coli* VF 3584). However, as shown by Figure 9, the four different concentrations of NMP only allows the range of NMP concentration where the optimum can be found but not a particular point-estimation. This is because the bi-linear model also has four parameters: $x_{\min}$, $x_{opt}$, $x_{\max}$, $y_{opt}$.

**Figure 10:**
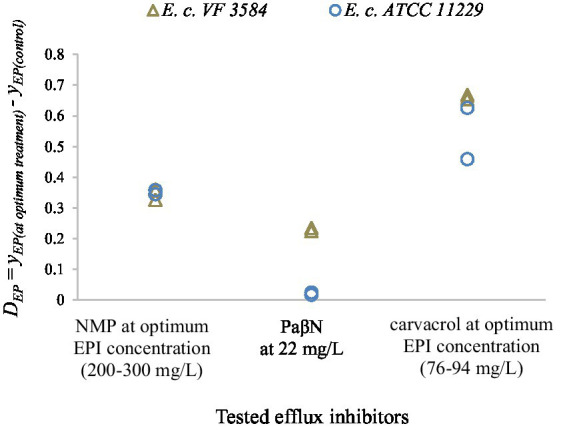
Difference between the respective *y*_*EP*_ values to parameters to compare the efflux modulation efficacy of the tested inhibitors (NMP, PaβN and carvacrol). Results for stationary phase cultures are represented by two independent replicates for *E. coli* VF 3584 (gray markers) and *E. coli* ATCC 11229 (blue markers). As a control, the *y*_*EP*_ values of untreated cultures were used.

As can be concluded, the optimum inhibitor concentration of NMP is lower for *E. coli* ATCC 11229 than for *E. coli* VF 3584 which is consistent with our previous finding with carvacrol. PaβN was tested only in 22 mg/l concentration. To compare the inhibitor efficacy of carvacrol with that of synthetic inhibitors, we created Figure 10, representing the differences between the respective *y*_*EP*_ values:


$$D_{EP}=y_{EP\left(at\;\text{optimum treatment}\right)}-y_{EP\;\left(\text{control}\right)}.$$

The results suggest that the inhibition efficacy of both NMP and PaβN were lower than that of carvacrol. At the optimum carvacrol treatment, the highest $y_{EP}$ values were measured which infer to the greatest inhibition on the accumulation of EtBr. PaβN seemed to be the least effective efflux inhibitor for these two *E. coli* strains; however, it must be said that this component was tested also at the lowest concentration.

## 4. Discussion

We showed experimental and numerical/statistical methods to determine the optimal concentrations of carvacrol, an efflux inhibitor. Traditional predictive microbiology methods were used to analyze data obtained by real-time fluorescence measurements.

Saturation function was fitted, as a primary model to fluorescence readings quantifying the EtBr accumulation within *E. coli* cells under a range of carvacrol treatments. Then the effect of carvacrol on a selected parameter of the primary model was described by a convex-from-below function to establish the optimum carvacrol concentrations for two *E. coli* strains at three physiological states. Finally, the effect of the physiological state of cultures on the estimated optimum concentration was analyzed. It was found that the membrane degradation effect of carvacrol increased with its concentration. Thus, while the primary model for membrane degradation was analogous (only a mirror image) to EtBr accumulation, the secondary model of membrane integrity measurements did not exhibit such an optimum. The chosen primary model parameter, the $y_{EP}=\ln\;(Fs_{1}/Fs_{0})$ ratio has the advantage that it is not sensitive to the initial values of the measured fluorescence curves. Although the estimates of the $y_{EP}$ parameter are rather noisy, the obtained primary model describes the expected pattern well and identified an optimum carvacrol concentration in the region of interest.

In our previous study, F-test showed that, even when two independent replicates produced different secondary models, the locations of their optima did not differ significantly. In the present study, F-test was used to show that the optimal carvacrol concentrations were very similar for the fast- and slow-growing cultures, while in the non-growing phase this optimum was higher (Table 1). We could not detect significant strain-effect on the optimum carvacrol concentrations. We used clinical and food isolates of *E. coli* as model organisms and found, for both strains, that EtBr failed to accumulate in the non-treated cells in the non-growing phase in the one-hour-long observation interval (Figure 8).

In the study of Whittle et al. (2021), the authors showed that there was no significant difference in EtBr accumulation when measuring it in single cells, at different growth phases. Only one case was different, when efflux deficient strains were used. The authors concluded that the reason for higher resistance in the stationary phase was not the lack of the AcrAB-TolC function, but the reduced permeability of the cells. The reduced permeability of stationary phase cultures can be formed to overcome the osmotic stress. During nutrient limitation, *E. coli* can lessen the permeability of the outer membrane, thus preventing the necessity for efflux (Mitchell et al., 2016; Jananee and Preeti, 2017).

However, our results, though at population level, indicated that the non-growing phase was a condition in which the bacterial stress response was general enough to excrete drugs from the cells. We found, for wild type *E. coli* strains, that the EtBr accumulation depends on the physiological state of the cells, both in the presence and absence of inhibitor.

At population level, the bacterial efflux activity could be increased as the response to the acidic environment due to the secondary metabolites in the both (Amaral et al., 2014; Nové et al., 2020). Since the efflux mechanism is a membrane-based system, it is not surprising that reduced susceptibility of bacteria in the non-growing phase was found. It can also be the result of induced resistance, which occurs when the target of the different stress factors is similar (Langsrud et al., 2004).

Our final predictive model can be used to optimize the application of other efflux inhibitors, too. For example, NMP, a synthetic inhibitor, was tested in four concentrations. Though this low number was not sufficient for point-estimation, we could identify the range of the optimum inhibitor concentration by our bi-linear model. The efflux inhibition efficacy of carvacrol was also compared to that of PaβN. We could conclude that carvacrol showed a remarkably higher efflux inhibition effect than either NMP or PaβN did.

We have expressed the optimal concentration of carvacrol as the proportion of its MIC value, to provide a more general overview of possible inhibitor concentrations. The concentrations were found in the range of 0.15–0.31 MIC values, where we observed a significant loss of EP activity *via* EtBr accumulation assays, and perceived that the membrane degradation is not significant yet. In this range, carvacrol only has mild antimicrobial activity, presumably not resulting in bacterial resistance.

Carvacrol, at high concentrations, makes pores on the lipid bilayer of the cytoplasmic membrane. This could well be the reason why we found that above a certain concentration of carvacrol, EtBr accumulation decreased. At high concentrations, EtBr could easily “escape” from the cells *via* porins. Beside the membrane disruption and efflux inhibition, other proposed mechanisms of carvacrol include the prevention of biofilm formation, chemotaxis and the inhibition of bacterial flagellar biosynthesis thus the motility (Yuan and Yuk, 2019; Boren et al., 2020). Another study proposed that carvacrol acts as a competitive NorA inhibitor (Dos Santos Barbosa et al., 2021). It was found that a single sub-inhibitory carvacrol treatment can decrease the expression of the MDR EP genes *MarA* and *AcrB* in *E. coli* O157:H7 (Yuan and Yuk, 2019). It was also shown that carvacrol is able to reduce the expression of proteins forming the AcrAB-TolC complex both in wild type and in mutant *E. coli* strains lacking one of the major efflux systems, AcrAB. Moreover, the strains in which *acrAB* genes were deleted and which were adapted to carvacrol had a lower antibiotic MIC than the parental strains that adapted also to carvacrol (Fadli et al., 2014). Yuan and Yuk (2019) demonstrated that meanwhile *E. coli* O157:H7 adapted to a sublethal carvacrol concentration, the motility, biofilm-forming ability, and EP activity of *E. coli* O157:H7 was reduced significantly using early stationary phase cultures. Although the periodic treatment resulted in upregulated multidrug efflux pumps (marA and acrB), the antibiotic resistance of bacteria was not induced. These and our finding also suggest that carvacrol is affecting the efflux system both directly and indirectly as it showed a concentration-dependent effect on the EtBr accumulation and the membrane integrity of bacteria as well.

In conclusion, carvacrol could be used as an efficient efflux inhibitor. It has all the desirable properties mentioned before: it is a non-toxic and safe agent and can be economically produced. Predictive modeling can help to find its optimal use, including the dependence of its optimum concentration on the physiological state of the cells. The conclusion, that the efflux mechanism depends also on the physiological state of the cells is an important addition to our general understanding of bacterial responses to stress.

## Data availability statement

The original contributions presented in the study are included in the article/supplementary material, further inquiries can be directed to the corresponding author.

## Author contributions

AJ, JB, AK, GK, and BS wrote this manuscript. AJ carried out the experiments. JB and AJ conducted the analyzes, and interpreted the data. AT-B and SSM were involved with original experimental design. All authors contributed to the article and approved the submitted version.

## Funding

This work was supported by the CEEPUS scholarship [grant numbers: CIII-HR-0306-11-1819-M-124303 – For Safe and Healthy Food in Middle-Europe] and funded by the ÚNKP-21-3-II New National Excellence Program of the Ministry of Innovation and Technology and the National Research, Development and Innovation Award and Slovenian Research Agency (ARRS J4-3088 and P4-0116).

## Conflict of interest

The authors declare that the research was conducted in the absence of any commercial or financial relationships that could be construed as a potential conflict of interest.

## Publisher’s note

All claims expressed in this article are solely those of the authors and do not necessarily represent those of their affiliated organizations, or those of the publisher, the editors and the reviewers. Any product that may be evaluated in this article, or claim that may be made by its manufacturer, is not guaranteed or endorsed by the publisher.

## Abbreviations

EtBr, Ethidium Bromide
EP, Efflux Pump
EPIs, Efflux Pump Inhibitors
Fs, Fluorescence signal
PBS, Phosphate Buffered Saline
RFU, Relative Fluorescent Unit
